# *Piece of Mind:* knowledge translation performances for public engagement on Parkinson’s disease and dementia

**DOI:** 10.3389/fpsyg.2024.1439362

**Published:** 2024-11-25

**Authors:** Naila Kuhlmann, Aliki Thomas, Natalia Incio-Serra, Stefanie Blain-Moraes

**Affiliations:** ^1^School of Physical and Occupational Therapy, McGill University, Montreal, QC, Canada; ^2^Center for Circus Arts Research, Innovation and Knowledge Transfer, National Circus School, Montréal, QC, Canada; ^3^Centre for Interdisciplinary Research in Rehabilitation of Greater Montreal (CRIR), Montreal, QC, Canada

**Keywords:** performing arts, circus, arts-based knowledge translation, lived experience, Parkinson’s disease, dementia, art-science, audience engagement

## Abstract

**Introduction:**

The subjective experience of illness is often overshadowed by the disease-and-cure focus of health research, contributing to the stigmatization of conditions such as Parkinson’s disease and dementia. This is exacerbated by the fact that traditional means of knowledge dissemination are inaccessible to non-academic audiences, hampering meaningful dialogue with and research uptake by the broader community.

**Methods:**

Our arts-based knowledge translation project, *Piece of Mind*, brought together neuroscientists, people with Parkinson’s disease or dementia, care partners and artists (musicians, dancers, circus acrobats) to co-create 2 multi-media performances based on scientific research and lived experience. We investigated whether the resulting interdisciplinary, multimedia performances could (1) challenge misperceptions around Parkinson’s/dementia; and (2) render neuroscientific research accessible to a diverse audience. Prior to and immediately following virtual screenings of the feature-length *Piece of Mind* Parkinson’s and Dementia filmed performances, audience members were invited to complete pre-post questionnaires comprised of demographic, Likert-scale and open-ended questions.

**Results:**

Responses indicated that both performances elicited strong emotional engagement and improved self-reported understanding and empathy towards individuals with Parkinson’s and dementia. Based on a thematic analysis on open-ended questions, we consider the barriers and facilitators to the audience’s receptiveness and discuss the performances’ potential as a knowledge translation tool.

**Discussion:**

By presenting an emotionally engaging perspective on Parkinson’s and dementia, *Piece of Mind* acts as an important complement to text-based knowledge dissemination in health research.

## 1 Introduction

Although self-report measures, clinical scales and qualitative inquiry are widely used in health research, they fail to capture *what it’s like* to live with a neurodegenerative condition such as Parkinson’s disease or dementia. This information is difficult to articulate at best; and at worst, impossible to access when verbal communication is directly impacted (Harasym et al., 2024). The disease-and-cure focus of health research further marginalizes the subjective experience of illness (Holmes et al., 2006; Wall, 2008), and traditional modes of knowledge production and dissemination limit engagement with non-academic stakeholders (Cummings et al., 2007; Grimshaw et al., 2012). Overall, a lack of meaningful dialogue between scientific research, lived experience and the general public contributes to misperceptions and stigma of Parkinson’s disease and dementia, threatening the social inclusion and support of people with neurodegenerative conditions (Dupuis et al., 2016; Kontos et al., 2020; Soundy et al., 2014; Tsiang and Woo, 2020; Wu et al., 2014). Disrupting current medical and public discourse on neurodegenerative conditions requires diversifying how we produce and communicate knowledge, enabling scientific and clinical research to enter in direct dialogue with the lived experience and contributing to more holistic understanding of persons living with these conditions (Dupuis et al., 2016; Stites et al., 2018).

The integration of arts-based approaches in health research has emerged as a promising strategy to foster a more inclusive, person-centered approach to health and illness (Boydell and Gladstone, 2012). The arts facilitate participatory research by opening points of entry to diverse stakeholders, thus capturing oft-overlooked facets of the phenomenon under study and allowing for perspectives from policy, advocacy and empowerment to influence the work (Majid and Kandasamy, 2021). Furthermore, harnessing the diverse forms of communication and expression conferred by artistic media allows us to extend beyond traditional form of knowledge translation (KT)—such as journal articles, research reports, policy briefs, presentations at scientific events—to reach new audiences and facilitate the co-production of knowledge and social change (Théberge et al., 2024). Performances that stage qualitative research findings have shown promise in reducing stigma, changing attitudes, and improving relational care towards persons with dementia (Genova, 2010; Gray, 2019; Jonas-Simpson et al., 2012; Kontos et al., 2020; Kontos and Naglie, 2007; Mitchell et al., 2006). More generally, previous studies have reported that art installations, theatre and dance can prompt healthcare practice modifications (Gray et al., 2003; Lapum et al., 2016; Mitchell et al., 2006) and policy changes (Leichner and Wieler, 2015) that better meet the needs of individuals living with neurological conditions.

The objective of the present study was to investigate whether co-created artistic performances that incorporate both scientific and experiential knowledge of neurodegenerative conditions would engage audiences on an emotional and intellectual level, and how these may confer novel perspectives and a different understanding than is possible in academic modes of research dissemination. We present the findings of an arts-based knowledge translation project entitled *Piece of Mind*, which brought together neuroscientists, persons living with neurodegenerative conditions, caregivers and artists to (1) facilitate knowledge exchange and promote empathy between these diverse stakeholders using participatory arts; and (2) co-create multi-media artistic performances to communicate scientific and experiential knowledge of Parkinson’s Disease (PD) and dementia. The objectives of the resulting performances were to (1) challenge public misperceptions of neurodegenerative conditions and (2) render neuroscientific research findings more accessible to the general public. Here, we report on the impact of the *Piece of Mind: Parkinson’s* and *Dementia* performances on audiences and demonstrate that high-quality arts-based KT can increase self-reported empathy, reduce stigma and misperceptions, and motivate behavioral change towards persons living with neurodegenerative conditions.

## 2 Materials and methods

### 2.1 Research approach

We present the findings of a mixed-methods study embedded in an arts-based knowledge translation project. The objective of this study was to investigate whether performances co-created by neuroscientific researchers, artists, people living with Parkinson’s disease or dementia and their caregivers could change viewers’ perceptions and attitudes towards these neurodegenerative conditions. Specifically, we aimed to determine the impact the performances had on the audiences’: (1) understanding of and curiosity towards the underlying scientific research; (2) understanding of and empathy towards people with lived experience; and (3) receptiveness to the use of performing arts as a vehicle for knowledge translation.

### 2.2 Procedure

#### 2.2.1 Co-creation of performances

*Piece of Mind: Parkinson’s* and *Dementia* were created in parallel by neuroscientists, artists, individuals living with PD and/or dementia and their caregivers (~20 participants per project). Through an iterative process of research and creation, we identified key messages to convey through the performances, and explored how knowledge from lived experience and scientific research could be translated using the performing arts. Group sessions (1–1.5 h; ~ 10 per project) were conducted over Zoom between November 2020 – April 2021. The sessions used dance and music to facilitate relationship-building, create common ground between participants, and explore issues around PD and dementia through creativity and embodiment. Performance themes were iteratively generated through improvisation, focused discussions, and small group collaborations, which resulted in music, choreography, poetry and theatrical scenarios. This creative content was synthesized over a 1-month artistic residency (June 2021) in a Montréal-based dance studio with the artistic directors, performers and production team.

The final multi-media performances included theatre, dance, circus, music, sound design and spoken word (Kuhlmann et al., 2023; Kuhlmann et al., 2024a). Audio clips from project participants were integrated throughout, and a participant living with PD acted as the main protagonist in the Parkinson’s performance. The performances were filmed and edited, resulting in two 45-min videos with multiple angles and effects. Additional resources were prepared to accompany the performances, including written scene descriptions, artists’ statements, and behind-the-scenes videos showing making-of footage and interviews with project participants. Further information on the process, as well as a qualitivate analysis of participant interviews, can be found in Kuhlmann et al. (2024b).

#### 2.2.2 Performance screening

The filmed performances and accompanying materials were launched on YouTube in July 2021 and remain freely available online.^1^ Four virtual screenings and talk-back sessions (2 English, 2 French) were hosted on Zoom, and both performances were screened for a Canada-wide high school summer program. The performances were advertised widely through social media, mailing lists, community partners, research conferences and word-of-mouth, primarily targeting the Montréal performing arts community, people affected by PD/dementia, healthcare professionals and scientific researchers.

### 2.3 Measures

Two questionnaires were devised to collect audience responses to the *Piece of Mind* *Dementia* and *Parkinson’s* performances (Supplementary material). The content was generated from arts-based KT and research-based theatre literature (Jonas-Simpson et al., 2012; Kontos et al., 2020; Kontos and Naglie, 2007; Kukkonen and Cooper, 2019; Mitchell et al., 2006; Rossiter et al., 2008b), and was reviewed by project participants, scientific experts and artistic experts. Viewer demographics and engagement with additional materials, provided on a website linked in the YouTube notes was assessed using multiple choice questions. Performance impact on awareness, empathy and knowledge of PD/dementia was assessed on a 7-point Likert scale. Open-ended questions were included to allow respondents to share impressions and feedback that could not be captured in close-ended questions (Kontos et al., 2020).

### 2.4 Data collection

Ethics was obtained from McGill’s Faculty of Medicine and Health Sciences Research Ethics Board. Audience members were encouraged to complete an online English or French pre- and post-questionnaire on Microsoft Forms (Supplementary material), which was sent out in advance to screening attendees and was linked in the YouTube video notes. A research statement was included at the beginning of the questionnaire and participants provided informed consent by submitting their responses. Participants had to have watched the corresponding *Piece of Mind* video and be able to respond to a French or English-language online questionnaire to participate in the study.

### 2.5 Data analysis

All data were managed in NVivo (version 12). Questionnaires were anonymized before analysis by assigning each one with a number and letter identifier. Responses to demographic questions were analyzed descriptively, and response frequencies were calculated for the Likert-scale questions. Author N.K. conducted a thematic analysis on the open-ended survey responses, using an inductive approach to assign descriptive codes to text segments (Braun and Clarke, 2006; Saldana, 2015). The codes were then sorted into broad categories, which were further refined into higher order themes. Through and inductive and iterative process, these themes were further reviewed for internal homogeneity (e.g., consistency) and external heterogeneity (ex. discrete themes), and their interrelationship was explored in a visual thematic map using the free Miro software (Braun and Clarke, 2006). Twenty percent of the surveys were independently coded by author N.I-S for consistency of interpretation of the data. All resulting codes and themes were discussed and revised with all authors at various stages of the analysis process.

## 3 Results

Between July 2021 and May 2024, the *Dementia* performance had been viewed 873 times on YouTube, and the *Parkinson’s* performance 1,586 times. At the time of our virtual screenings, 125 respondents completed the pre-performance questionnaires (*Dementia n* = 75 English, 5 French; *Parkinson’s n* = 27 English, 18 French), and 59 completed the post-performance questionnaires (*Dementia n* = 29 English, 4 French; *Parkinson’s n* = 13 English, 13 French). The respondents’ demographic characteristics are summarized in Table 1.

**Table 1:** Demographic characteristics of study participants.

Characteristic	Parkinson’s respondents; No. (%) (*N* = 45)	Dementia respondents; No. (%) (*N* = 80)
**Gender**		
Man	12 (26.7)	26 (32.5)
Woman	31 (68.9)	50 (62.5)
Non-Binary	1 (2.2)	2 (2.5)
Other	0 (0)	0 (0.0)
Prefer not to say	1 (2.2)	2 (2.5)
**Age Group**		
< 20 years	0 (0)	53 (66.3)
21–35	15 (33.3)	8 (10.0)
36–50	10 (22.2)	11 (13.8)
51–65	11 (24.4)	6 (7.5)
66–80	7 (15.6)	1 (1.3)
> 80	1 (2.2)	1 (1.3)
Prefer not to say	1 (2.2)	0 (0.0)
**Experience with Parkinson’s disease (PD)**		
I have Parkinson’s disease.	6 (13.3)	N/A
I am a family member of someone with PD.	9 (20.0)	N/A
I am a friend of someone with PD.	7 (15.6)	N/A
I am a graduate student researching PD.	3 (6.7)	N/A
I am a professor researching PD.	0 (0.0)	N/A
I am a healthcare professional caring for people with PD.	1 (2.2)	N/A
I am a complementary therapist (ex. music, dance, art, etc.) caring for people with PD.	0 (0.0)	N/A
I have no connection with PD.	17 (37.8)	N/A
Other	4 (8.9)	N/A
**Experience with dementia**		
I have dementia.	N/A	0 (0.0)
I am a family member of someone with dementia.	N/A	28 (35.0)
I am a friend of someone with dementia.	N/A	2 (2.5)
I am a graduate student researching dementia.	N/A	2 (2.5)
I am a professor researching dementia.	N/A	1 (1.3)
I am a healthcare professional caring for people with dementia.	N/A	1 (1.3)
I am a complementary therapist (ex. music, dance, art, etc.) caring for people with dementia.	N/A	2 (2.5)
I have no connection to dementia.	N/A	42 (52.5)
Other	N/A	10 (12.5)

**Figure 1:**
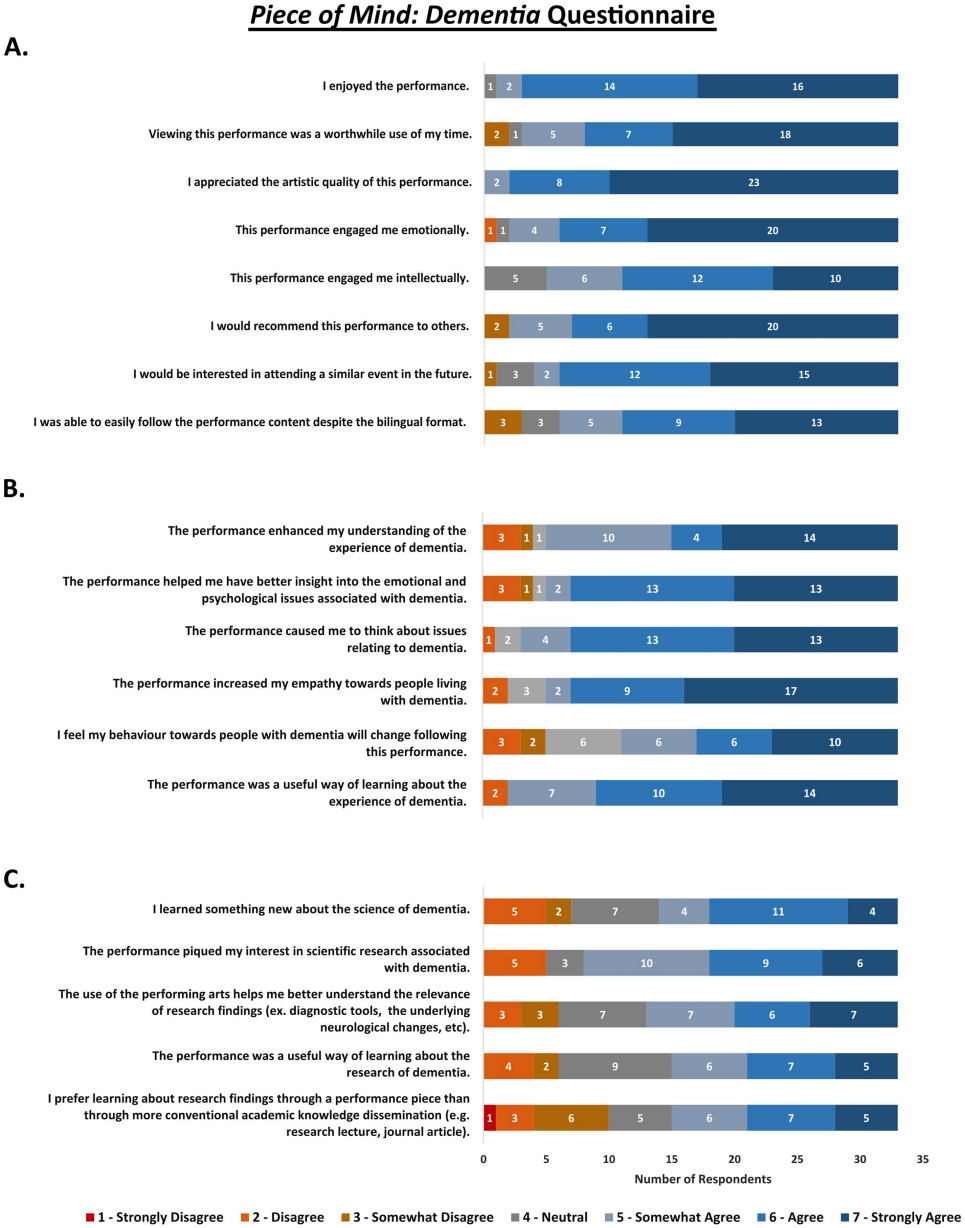
*Piece of Mind*: *Dementia* close-ended questions and responses. Combined results from English- and French-language post-performance questionnaires (*n* = 33 respondents), regarding the general enjoyment of the performance (A), and the reception of the content based on the lived experience (B) and scientific research (C) of dementia.

**Figure 2:**
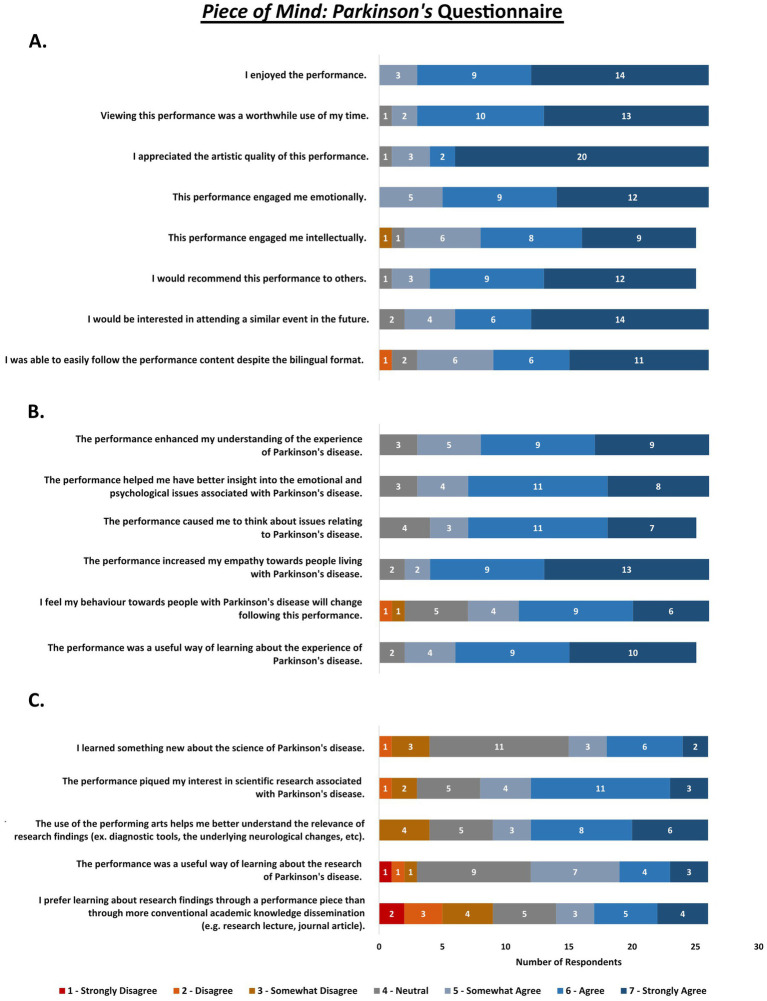
*Piece of Mind*: *Parkinson’s* close-ended questions and responses. Combined results from English- and French-language post-performance questionnaires (*n* = 26 respondents), pertaining to the general enjoyment of the performance (A), and the reception of the content based on the lived experience (B) and scientific research (C) of Parkinson’s disease.

**Figure 3:**
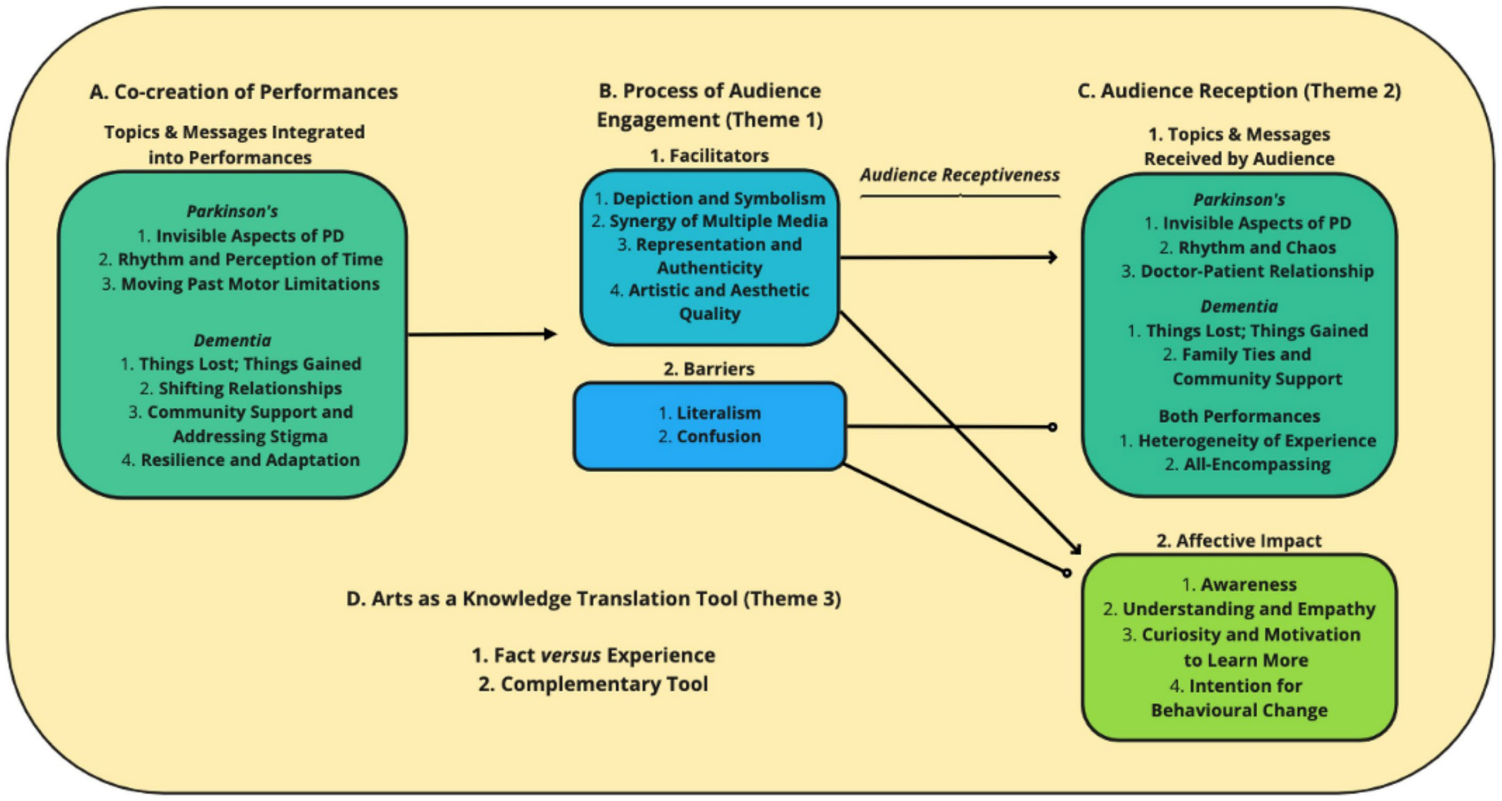
Inputs, process and outcomes of audience receptiveness to *Piece of Mind* performances. An illustration of how the key topics and messages integrated into *Piece of Mind: Parkinson’s* and *Dementia* were received by the audience, and the identified facilitators and barriers that determined the spectators’ receptiveness and the resulting affective impact of the performances. (A) The key messages to convey and topics to highlight were identified and agreed upon by participants during the co-creation process, and were intentionally woven throughout the scenes of each performance. (B) Based on responses to open-ended questions, we determined elements of the performances that appeared to facilitate (B1) or impede on (B2) the viewers’ receptiveness to both the informative and emotional content (C). (C1) Respondents were asked which key messages they took away from the performances, and additional references to these were coded throughout the responses. The key messages emerging from viewers’ responses were slightly altered, but consistent with, those that went into the performances. (C2) Several affective reactions to the performances emerged from the questionnaire responses, both from strong agreement with related Likert-scale questions, and from respondents’ describing the impact of the viewing experience in their own words. (D) The respondents’ comments on their viewing experience the reception of the performances allowed us to reflect on the affordances and limitations of the arts as a KT tool, particularly with regards to conveying factual information versus lived experience (D1), and in acting as a complement to traditional research dissemination and other KT strategies (D2).

The responses to close-ended questions regarding the *Piece of Mind* performances are summarized in Figure 1 (*Dementia*) and Figure 2 (*Parkinson’s*). Data from the open-ended responses were organized into three themes: (1) the process of audience engagement; (2) audience reception; and (3) arts as a KT tool. Each theme contained multiple sub-themes described below, with an overview provided in Figure 3.

### 3.1 Process of audience engagement

Open-ended questions prompted respondents to detail how various elements of the performances impacted their viewing experience. These allowed us to identify potential facilitators (3.1.1) and barriers (3.1.2) to the audiences’ receptiveness to and emotional engagement with the performances’ content.

#### 3.1.1 Facilitators to viewers’ receptiveness

##### 3.1.1.1 Depiction and symbolism

Most respondents (*n* = 37) highlighted evocative imagery as a key facilitator of their receptiveness. Many commented that their emotional response to, and understanding of, the performances’ content was enabled by depiction, metaphor, and symbolism. Some identified specific scenes as particularly visually captivating or symbolic, while others noted that seeing the information portrayed on stage struck a different chord than reading it in a scientific article. One viewer of the *Parkinson’s* performance reflected:

“It is hard to relate to the lived experiences if you have never experienced the deficits. Especially for people without PD, it is easy to understand that it would be ‘inconvenient’ and different for the patients to live with motor deficits, but it is hard to grasp what deficits such as bradykinesia, resting tremor, etc. can feel like. By seeing dancers change the speed, direction, and sharpness of their movements according to the background music, it made it easy to understand what it would be like to have these motor deficits.”

##### 3.1.1.2 Synergy of multiple media

Given the complexity of the experiences we wished to capture on stage, we made a deliberate choice to combine a variety of media (e.g., dance, music, circus, spoken word) and artistic genres. Fifteen participants specifically noted the diversity of media used and the synergy between them as a strength of the performances.

The use of multiple artistic media additionally allowed for a layering of content, adding dimension and entry points into the experiences and information being communicated. One viewer remarked how this made the representation of dementia more holistic, particularly in the inclusion of audio samples from our group sessions:

“This performance was so different in terms of the presentation, music, choreography, lighting, and voice-overs, in a really good way. I believe that without these added elements that were created as a result of the many Zoom calls and the wide variety of technologies that we now possess, this film would not have been as impactful and truthful to how patients themselves experience dementia. Thus, I really appreciate how this performance incorporated many elements that made scientific knowledge connect to real life experiences.”

##### 3.1.1.3 Representation and authenticity

An important element of the performances was how believable they felt to the viewer—particularly in representing the lived experience of people living with neurodegenerative conditions. Twenty-three respondents highlighted that the credibility of the performers, the recorded participant testimonials, and the presence of a person with PD on stage for *Parkinson’s* made it feel authentic. With regards to the latter, one viewer remarked:

“Having a patient being part of the performance is vital! […] It was very interesting to hear the “real” patients talk about their experiences, their frustrations, their struggles… I found it a perfect combination to the performance because it helped me dig deeper in the disease [*sic*] through first-hand insights.”

##### 3.1.1.4 Artistic and aesthetic quality

Over 70% of respondents across both performances marked “Strongly Agree” in response to *“I appreciated the artistic quality of this performance”* (Figures 1A, 2A). Viewers praised the skill and expressivity of the performers, the beauty of the music or visual elements, the narrative arch and seamless transitions, and the quality of the filming and production. With regards to the strengths of the *Parkinson’s* performance, one viewer noted,

“Variety of artistic practices performed at a high level (music, circus, different types of dance), creative set pieces, poignant interpretation of delicate subjects, great runtime (not too short, not too long), great final act (great ending).”

#### 3.1.2 Barriers to viewers’ receptiveness

##### 3.1.2.1 Literalism

Moments in which information was conveyed didactically, with less artistic interpretation, detracted from the performances. Although this was only explicitly stated in a handful of responses (*n* = 6), some viewers—particularly for *Parkinson’s*—listed the scenes focused on scientific content as a weak point, remarking they were “heavy,” “dense” or “stilted.” This led viewers to feel that the “intellectual and artistic content distracted from each other at times.” In reference to a scene depicting some of the neurobiological changes underlying PD, one viewer noted a lack of integration between the scientific knowledge and lived experience:

“Regarding the artistic aspect, there was some loss of connection between what happens in the brain and how one feels. I saw what happens to different neurotransmitters as a result of the disease, but I did not see how that leads to the experienced symptoms. […] That part was pretty information heavy.”

##### 3.1.2.2 Confusion

Twenty-one respondents identified confusion as a barrier to their receptiveness. This was primarily in response to *Dementia*, where certain choreographies were purposefully more abstract and open to interpretation. However, some viewers reported uncertainty about whether the evoked confusion was intentional or not, which distracted from the content at times. One respondent emphasized the use of dance as a source of confusion, noting:

“I think that some scenes with the dance moves were a bit difficult for me to understand, as the different positions looked similar to me. Maybe it is because of my inexperience with dance, but I think that some moves may have meant to convey certain complex emotions or feelings that I missed out from.”

### 3.2 Audience reception

Audience reception was sub-divided into the effectiveness of knowledge transfer, e.g., the messages respondents reported receiving from the performances (3.2.1), and the affective impact on viewers (3.2.2).

#### 3.2.1 Messages conveyed through the performances

##### 3.2.1.1 Piece of Mind: Dementia

Fourteen respondents noted the impact of dementia on family and close relationships, and the importance of continuing to provide love and support to the person with dementia (*Family Ties & Community Support*). As one respondent put it,

“I think the key message that I took from the performance is that due to the stigma associated with dementia along with the difficulties and frustration that those with dementia face, it is incredibly important for their family and friends to provide love and support.”

The message of *Things Lost; Things Gained* was also clearly received by audiences; while sadness, confusion, frustration, and grief were highlighted as key aspects of the dementia experience, many viewers (*n* = 11) also commented on the resilience, adaptation and silver linings that were apparent within the performance.

##### 3.2.1.2 Piece of Mind: Parkinson’s

The *Invisible Aspects* of PD was effectively conveyed in the *Parkinson’s* performance, with twelve respondents noting how much of the disease experience extends past what meets the eye. Viewers highlighted how the performance captured the inner experience of illness, and that “[…] what is visible is not everything about living with Parkinson’s disease.” More specifically, six respondents listed “chaos” as a feeling evoked by the performance, and three noted how PD changes rhythm and time perception (*Rhythm & Chaos*).

Nine viewers indicated the performances’ depiction of the doctor-patient relationship as an important take-away. In reflecting on the “Navigating the Medical World” scene, one respondent noted,

“The stark difference between how the PD patient in the performance responded to her changes versus the two doctor’s observations and commentary on the patient was very shocking and powerful moment. Although the physicians and researchers of PD are often “most” knowledgeable about the causes, symptoms and scientific backgrounds of PD, because they do not live with PD, they do not perceive PD the same way the patients do in their everyday lives.”

Although *Moving Beyond Motor Limitations* was not explicitly identified in audience responses, many expressed an increased appreciation for the motor complications and admiration of the resilience displayed in the performance.

##### 3.2.1.3 Both performances

Two additional take-aways emerged in the analysis of viewers’ responses across both performances. The first was the *Heterogeneity of Experience*, with nine respondents noting how different both the manifestation and experience of PD and dementia can be from one individual to the next and highlighting that the disease experience is “very much [one’s] own.” After viewing the *Dementia* performance, one respondent reflected,

“Dementia is not always clear-cut, nor is it the same for everyone. This condition will affect everyone differently, and it is hard for a patient with dementia to convey their message to the world as their brains are affected by many factors that changes their perception of the world and information.”

The *All-Encompassing* nature of living with PD or dementia was also emphasized, with ten respondents expressing that the performances made them realize the far-reaching consequences of these conditions. As one viewer of the *Parkinson’s* performance remarked:

“The lived experiences of the patients exceed beyond medically described motor deficits and impact every portion of daily lives and activities.”

#### 3.2.2 Affective impact of performances

In addition to delivering intellectual content, both performances impacted the audience emotionally: they fostered awareness, empathy and understanding, incited curiosity to learn more about neurodegenerative conditions, and created an intention to change behaviors towards persons with neurodegenerative conditions.

##### 3.2.2.1 Receptiveness to being moved

The concept of receptiveness was an important finding based on the frequency with which the performances were described as “powerful,” “engaging,” “captivating,” “moving,” and “touching.” One participant expressed that the feeling of confusion “was so powerful that it is still vivid in my body at the moment”; another that the performance on dementia “moved [them] to think about it differently.” This was supported by overwhelmingly positive responses on the corresponding Likert-scale question regarding emotional engagement (Figures 1A, 2A), and the range of emotional descriptors reported when asked what feelings the performances evoked.

This engagement was such that one respondent noted that he/she “sometimes […] even forgot about the theme just to focus on the emotion that the show evoked.” That said, it acted as an important vehicle for engaging with the performances’ content; as one viewer put it, “experiencing the emotions allowed me to connect and sympathize better with the story.”

##### 3.2.2.2 Awareness

Nearly half of respondents (*n* = 25) expressed greater awareness of the experiences of, and issues surrounding, PD and dementia following the performances. While many participants explicitly stated this as an outcome, others alluded to it by expressing that the performances were “awakening,” or that they caused a shift in perspective regarding issues around PD/dementia. After viewing the *Dementia* performance, one respondent shared,

“Originally, I thought that dementia only affected patient’s memories and emotions, but after seeing the performance, it makes my understanding of dementia much more whole. Instead of only grasping a few ideas before I watched this performance, now I can see how dementia affects daily lives and how when little details that may seem ‘insignificant’ to others without dementia are changed, this could dramatically change the lives of the patients with dementia.”

Another respondent provided a concrete example of how the *Parkinson’s* performance impacted his/her perceptions and reflections in a real-life context:

“Just yesterday I went to eat in a restaurant, and I think the person next to me was living with Parkinson’s disease: companion dog, tremor, rigid facial expression. Right away I thought about the performance in the show; I saw beyond the person sitting beside me with a caregiver. I could imagine his/her day-to-day a little better, knowing it differs for everyone.” (Translated from French).

##### 3.2.2.3 Empathy and understanding

Most viewers (*n* = 35) described feeling more empathy for, and having a deeper understanding of, PD and dementia after viewing the performances. Many expressed this as an increased ability to connect or relate to people living with these conditions. The performances were praised for their humanness; one viewer noted that “it helped [them] to better see the person living with the disease instead of just the disease” (*Dementia*), while another expressed a “new appreciation of PD from the eyes of the patient.” (*Parkinson’s*).

The self-reported empathy was often stated in combination with a more visceral emotional response, as in the words of a viewer of the *Dementia* performance:

“It evoked empathy and sadness for me. It was heartbreaking to see some of the more somber parts of the performance, but I do think it helped me understand the relationship dynamics between people with dementia and their friends and family better.”

Similarly, a viewer of the *Parkinson’s* performance listed numerous emotional reactions, noting:

“The performances evoked feelings of ‘loss of control’, helplessness, confusion, frustration, and the patient’s desire to run away from the situations. Watching the artistic expression of the lived experiences through dancers and music were more helpful and personally touching than reading the descriptions of the motor deficits. It was easy to relate to what it could feel like.”

This was supported by most respondents answering “Somewhat Agree” or higher on the Likert-scale questions about the performances’ ability to convey lived experience (Figures 1B, 2B).

##### 3.2.2.4 Intention for behavioral change

Both the Likert-scale and open-ended responses suggested that the performances elicited an intention for behavioral change towards persons with neurodegenerative conditions. Over 62 and 73% of respondents marked “Somewhat Agree” or higher in response to a statement regarding behavioral change following the *Dementia* (Figure 1B) and *Parkinson’s* performance (Figure 2B), respectively. In the written responses, one-third (*n* = 20) of participants expressed that they intended to change their behavior towards persons with neurodegenerative conditions, and to share the performance with others. In relation to his/her profession, one viewer stated,

“I think I will not assume that if someone with PD looks ok, that they feel ok. I’ll try to make sure to check in with people more regularily to see that they are good with the activities we are doing.”

##### 3.2.2.5 Curiosity to learn more

Sixteen participants reported having learned factual information from the performances, or a desire to know more about PD/dementia. Many of these comments referred to the performances “sparking curiosity” or “piquing interest,” and a motivation to consult our additional resources provided on a website accompanying the performance, or to read more about these conditions. While Likert-scale responses were less positive regarding the ability to convey scientific content (Figures 1C, 2C), most respondents nonetheless answered “Somewhat Agree” or higher to statements regarding the performances’ ability to prompt curiosity and support learning. The motivation to learn was not only pertaining to known scientific facts about these conditions; as one viewer put it,

“It [the performance] made me realize how important it is as a researcher, doctors, and as a community, we need to extend beyond looking at PD as a disease and learn how to support PD patients holistically.”

### 3.3 Arts as a knowledge translation tool

The audiences’ responses to the performances illuminated two main findings regarding the affordances and limitations of arts-based KT: its ability to convey fact and experience (3.3.1), and its place as a complementary tool to traditional modes of research dissemination (3.3.2).

#### 3.3.1 Factual versus experiential knowledge

While the performances illustrated the “human aspect” of PD/dementia, respondents critiqued their ability to communicate fact-based knowledge (*n* = 23). There were mixed opinions regarding the importance of the latter in arts-based KT: some wanted more explanation of the scientific research; others preferred foregrounding the human experience in a multimedia performance. The tension between giving place to both experiential and scientific knowledge in the same performance is best captured by one respondent’s reflections:

“I feel that I was somewhat engaged intellectually throughout the performance and would have been even impressed with the performance engaged me even more. However, I also understand that it is hard to balance between inserting more explicit scientific knowledge versus letting the artistic expressions implicitly explain itself. […] While I feel that this performance is excellent for understanding the emotional and physical aspects of living with PD for people who are not familiar or those who know scientific background of PD but not the lived experiences, I do feel that there are some limitations expressing scientific research findings through a performance.”

In considering what audiences look for in a performance, another respondent wrote,

“I do not think most people are looking for information “about” dementia so much as they are looking for validation of their feelings around it, and perhaps some practical advice for what to do. I do not think performance (at least not this performance) is a great medium for practical tips, but there’s lots of value in building a shared experience and giving people a way to explore those feelings outside of direct experience with a loved one. […] I think the biggest value is the chance that a multimedia performance can build cultural practices around dementia that do not push those who are coping with it into a corner out of sight.”

These qualitative findings were supported by the Likert-scale responses, with positive statements regarding the lived experience aspect of both performances (Figures 1B, 2B) receiving stronger agreement than those pertaining to the scientific content (Figure 1C).

#### 3.3.2 Complementary tool

The multi-media approach was praised as innovative and effective in adding an emotional component that is generally lacking from other means of scientific communication (e.g., research articles, conferences), and in being more engaging for a wide audience (*n* = 17). Respondents noted that while arts-based KT cannot replace other methods, it is an important example of diversifying dissemination channels. In the words of one viewer, an artistic performance “provides more insight on the lived experience of dementia than reading an article about it would”; however, the latter is important to “eliminate the possibility of misinterpreting what dementia is.” Echoing this sentiment, another viewer concluded that an artistic performance “works best as a source of examples and metaphors that can be explored in conjunction with other learning tools (e.g., lecture, discussion, supplemental reading).”

## 4 Discussion

Although the past decades have seen significant advances in the etiology, management and treatment of neurodegenerative conditions, the focus on clinical outcomes has distorted the image of aging (Inouye et al., 2021). Our work demonstrates the ability of KT artistic performances to provide an important complementary viewpoint of aging-related illnesses, both by integrating the perspectives of diverse knowledge creators, and by entering in dialogue with non-academic audiences. We illustrate that multi-media performances can allow for biological, psychosocial and phenomenological topics to share the stage, thereby presenting a more complete, person-centered picture of aging.

*Piece of Mind*’s use of artistic media was evocative and effective in conveying the intended messages, and moved audience members to engage with the issues presented. The questionnaire responses highlighted that our performances were successful in raising awareness, fostering empathy and addressing stigma concerning PD/dementia. These findings align with those from previous arts-based endeavors to convey the phenomenology of illness, and add to the growing literature of the unique affordances and limitations of arts-based KT in healthcare (Boydell et al., 2016; Boydell, 2011; Colantonio et al., 2008; Deloney and Graham, 2003; Gray, 2009; Gray and Sinding, 2002; Jonas-Simpson et al., 2012; Kontos et al., 2020; Lafrenière and Cox, 2013; Lapum et al., 2012; Leichner and Wieler, 2015; Rieger et al., 2021; Rossiter et al., 2008a; Rossiter et al., 2008b).

Previous research-based dramas about dementia have similarly reported a strong emotional impact on general audiences (Mitchell et al., 2006), meaningful changes in healthcare professionals’ views of dementia (Jonas-Simpson et al., 2012), and acknowledgement of the importance of tacit and embodied knowledge in providing person-centered care (Kontos and Naglie, 2007). Although tracking concrete behavioral outcomes of artistic performances is difficult (Parsons et al., 2017), numerous frameworks have been proposed to evaluate arts-based KT and the mechanisms by which audiences are moved to think or act differently (Kontos and Poland, 2009; Lafrenière and Cox, 2013; Parsons et al., 2017; Radbourne et al., 2009; Rieger and Schultz, 2014). These converge on the importance of (1) aesthetic quality, (2) a truthful and meaningful depiction of the research, and (3) inviting audiences to engage with the content in an active and iterative manner (Lafrenière and Cox, 2013; Parsons et al., 2017; Rieger and Schultz, 2014). Despite challenges in balancing artistic decisions with the accurate representation of research findings (Boydell et al., 2016; Rieger and Schultz, 2014), providing an aesthetic lens through which viewers can approach the latter can elicit emotional engagement (Colantonio et al., 2008; Lapum et al., 2012) and immersion that enables transformative forms of learning (Kontos et al., 2020). Concurrently, accurately representing the source information is critical for the effectiveness of arts-based KT (Bagley, 2009; Colantonio et al., 2008; Lafrenière and Cox, 2013), as the perceived trustworthiness of the depiction impacts a viewer’s degree of engagement (Radbourne et al., 2009). This was reflected in our respondents’ focus on the performances feeling “real” and resonating with their own experiences, which was also noted as a key factor in the reception of *Cracked* (Kontos et al., 2020) and in the integrated of patients’ voices in an artistic installation on congenital heart disease (Biglino et al., 2019). Similarly, Mitchell and colleagues have stated that truth was the “linchpin” in determining whether or not their play was received as a valuable representation of the experience of dementia (p. 202) (Mitchell et al., 2006); they describe the synergies that occur when a performance’s content is deemed authentic, allowing the viewer to feel transported into the scenes and to subsequently be moved by them (Mitchell et al., 2011). Depiction and symbolism can bring information to life in a new way on stage, allowing for a more complex and evocative representation than is possible in text (Gray and Kontos, 2015; Kontos and Poland, 2009). Not only did the synergy between different artistic media in our work enable multiple modes of engagement with the subject matter for diverse audiences (Novy et al., 2021), but this multi-faceted approach is arguably essential for fostering a sense of “emotional truth” (Rossiter et al., 2008b).

Overall, the aesthetic merit, rich depiction, and authenticity of the work contribute to the viewer’s *receptiveness,* or *resonance*: “[…] research’s ability to meaningfully reverberate and affect an audience.” (p. 844) (Tracy, 2010). Thus, while academic articles are essential for communicating new evidence within a research and clinical context, arts-based KT can convey the feelings, nuances and relevance of these facts to everyday experiences (Rieger and Schultz, 2014), and challenge audiences to reconsider their preconceived notions of neurodegenerative conditions.

Despite the promise of arts-based KT as a tool for enhancing aging and challenging agism (Kontos and Naglie, 2007; Rieger et al., 2021; Rossiter et al., 2008b; Shapiro and Hunt, 2003), it is not suited for all purposes (Colantonio et al., 2008). Arts-based approaches lend themselves more naturally to subjective experiences and embodied forms of knowledge (Boydell and Gladstone, 2012; Kontos and Naglie, 2009; Rieger and Schultz, 2014), and our work contributes to a growing sci-art movement aiming to render neuroscientific research more captivating for general audiences (Zaelzer, 2020). While our performances piqued curiosity, they were not conducive to directly conveying factual information—perhaps due to a conflict between the literalism employed and the abstraction necessary for emotional immersion (Gray, 2009; Gray and Kontos, 2015). Arts-based approaches may be particularly effective for *experiential learning* (Deloney and Graham, 2003; Jonas-Simpson et al., 2012) and for the affective—but not cognitive—domain of Bloom and Krathwohl’s hierarchy of learning (Krathwohl et al., 1964; Krathwohl, 2002). We thus consider our performances as an invitation to learn more, and provide additional resources for the viewer to explore the presented information in greater detail (Radbourne et al., 2009). Given the boundaries of arts-based KT, in addition to high production costs, the inherent trade-offs, and the difficulty in evaluating impact (Parsons et al., 2017; Rossiter et al., 2008a; Rossiter et al., 2008b), these strategies should be treated as an important complement to—rather than replacement for—other forms of KT (Graham et al., 2006; Grimshaw et al., 2012; Rycroft-Malone et al., 2015). Indeed, arts-based KT may be particularly well-suited to promoting practice and policy changes where academic research dissemination strategies fall short (Théberge et al., 2024), could be integrated into medical education to diversify learning approaches and promote empathy, and provides a powerful support and source of empowerment to individuals personally affected by neurodegenerative conditions.

## 5 Limitations and future directions

Our findings need to be interpreted in light of several limitations. First, virtual data collection in response to COVID-19 reduced our sample size, as viewers were less likely to respond to an online questionnaire. This also increased the selection bias in our respondents, as the most invested viewers were the most likely to fill out the questionnaires. Second, the Likert-scale and open-ended questions that were most appropriate for online data collection did not permit a thick analysis of participant reactions; future research would benefit from using focus groups and standardized scales of empathy with more diverse, live audiences. Finally, the versions of *Piece of Mind* available online are static and not able to shift with audience feedback. Having since had the opportunity to present live performances and excerpts in both academic and artistic settings, we continue to build on the work, allowing viewers’ interpretations and feedback to guide the evolution of our performances and using our research findings to inform their adaptation to new contexts and target audiences.

## 6 Conclusion

By presenting an accessible and emotionally engaging perspective on the research and lived experience of PD/dementia, *Piece of Mind* contributes to addressing stigma towards, and promoting social support for, people living with these conditions. As knowledge production and dissemination on healthy aging continues to be dominated by academic researchers, arts-based strategies such as our own offer a unique opportunity for diverse stakeholders to voice alternative disease narratives, and for audiences to reflect on neurodegenerative conditions in a new light.

## Data Availability

The raw data supporting the conclusions of this article will be made available by the authors, without undue reservation.

## Footnotes

^1^ www.youtube.com/@pieceofmindcollective9587
