# Supplementary material for: Piece of Mind: knowledge translation performances for public engagement on Parkinson’s disease and dementia
Source: Front Psychol. 2024 Nov 25;15:1439362. doi: 10.3389/fpsyg.2024.1439362 (PMC11627042; doi:10.3389/fpsyg.2024.1439362)
Supplement: Supplementary file 1 [file Data_Sheet_1.docx]

**Supplementary Methods**

1. *Parkinson’s* and *Dementia* pre-performance questions (English)

2. *Parkinson’s* and *Dementia* post-performance questions (English)

3. *Parkinson’s* and *Dementia* pre-performance questions (French)

4. *Parkinson’s* and *Dementia* post-performance questions (French)

**Note**: questionnaires for both performances were identical, except for the use of “Parkinson’s disease” or “dementia” within the questions.

**1. *Parkinson’s* and *Dementia* pre-performance questionnaire (English)**

**1. How would you best describe your connection to [Parkinson’s disease / dementia]? Please check all that apply.**

- I have Parkinson’s disease / dementia
- I am a family member of someone with Parkinson’s disease / dementia
- I am a friend of someone with Parkinson’s disease / dementia
- I am a graduate student researching Parkinson’s disease / dementia
- I am a professor researching Parkinson’s disease / dementia
- I am a healthcare professional caring for people with Parkinson’s disease / dementia
- I am a complementary therapist (ex. music, dance, art, etc.) caring for people with Parkinson’s disease / dementia
- I have no connection to Parkinson’s disease / dementia
- Other : _________

**2.** **Which best describes your gender?**

- Man
- Woman
- Non-Binary
- Other: ________
- Prefer not to say

**3. What is your age?**

- < 20
- 20 – 35
- 35 - 50
- 50 – 65
- 65 – 80
- > 80
- Prefer not to say

**4.** **Do you understand both French and English?**

- English only
- Both, but prefer French
- Both, but prefer English
- Both, equally

**5. On a scale of 1 to 7, to what extent do you understand the lived experience of Parkinson’s disease / dementia?**

1 = Not at all
2 = Very Poorly
3 = Poorly
4 = Somewhat
5 = Well
6 = Very well
7 = I fully understand it

**6. On a scale of 1 to 7, to what extent do you understand the scientific research of Parkinson’s disease / dementia?**

1 = Not at all
2 = Very Poorly
3 = Poorly
4 = Somewhat
5 = Well
6 = Very well
7 = I fully understand it

**7. Please feel free to share a few sentences about why you are interested in this performance, and what you hope to get from it.**

**2. *Parkinson’s* and *Dementia* post-performance questionnaire (English)**

***Open-Ended Questions:***

**1. What key messages did you take from the performance?**

**2. What feelings did the performance evoke? What was it like to experience it?**

**3. What were some strengths of the performance?**

**4. What were some weaknesses of the performance?**

**5. ﻿** **Will this performance change how you think about or act regarding Parkinson’s disease / dementia? How?**

**6. Do you think a multimedia performance is an appropriate format to learn about both the lived experience and scientific research of [Parkinson’s disease / dementia]? Please explain.**

***Enjoyment of performance***

Please use the following scale in answering the questions below.
1 = Strongly disagree ; 2 = Disagree ; 3 = Somewhat Disagree ; 4 = Neither Agree nor Disagree ; 5 = Somewhat Agree ; 6 = Agree ; 7 = Strongly Agree

**7. I enjoyed the performance.**

1 2 3 4 5 6 7

**8. Viewing this performance was a worthwhile use of my time.**

1 2 3 4 5 6 7

**9. I appreciated the artistic quality of this performance.**
1 2 3 4 5 6 7

**10. This performance engaged me emotionally.**

1 2 3 4 5 6 7

**11. This performance engaged me intellectually.**

1 2 3 4 5 6 7

**12. I would recommend this performance to others.**

1 2 3 4 5 6 7

**13. I would be interested in attending a similar event in the future.**

1 2 3 4 5 6 7

**14. I was able to easily follow the performance content despite the bilingual format.**

1 2 3 4 5 6 7

**15. Please feel free to elaborate on any of your answers above.** (open-ended question)

***Lived experience***

Please use the following scale in answering the questions below.
1 = Strongly disagree ; 2 = Disagree ; 3 = Somewhat Disagree ; 4 = Neither Agree nor Disagree ; 5 = Somewhat Agree ; 6 = Agree ; 7 = Strongly Agree

**16. The performance enhanced my understanding of the experience of Parkinson’s disease / dementia.**1 2 3 4 5 6 7

**17. The performance helped me to have better insight into the emotional and psychological issues associated with Parkinson’s disease / dementia**.

1 2 3 4 5 6 7

**18. The performance caused me to think about issues relating to Parkinson’s disease / dementia in new ways.**

1 2 3 4 5 6 7

**19.** **The performance increased my empathy towards people living with Parkinson’s disease / dementia.**

1 2 3 4 5 6 7

**20. I feel my behaviour towards people with Parkinson’s disease / dementia will change following this performance.**

1 2 3 4 5 6 7

**21. The performance was a useful way of learning about the experience of Parkinson’s disease / dementia.**

1 2 3 4 5 6 7

**22. Please feel free to elaborate on any of your answers above.** (open-ended question)

***Scientific communication***

**23. I learned something new about the science of Parkinson’s disease / dementia.**

1 2 3 4 5 6 7

**24. The performance piqued my interest in scientific research associated with Parkinson’s disease / dementia.**

1 2 3 4 5 6 7

**25. The use of the performing arts helps me better understand the relevance of research findings (ex. diagnostic tools, the underlying neurological changes, etc.).**

1 2 3 4 5 6 7

**26. The performance was a useful way of learning about the research of Parkinson’s disease / dementia.**

1 2 3 4 5 6 7

**27. I prefer learning about research findings through a performance piece than through more conventional academic knowledge dissemination (e.g. research lecture, journal article).**

1 2 3 4 5 6 7

**28. Please feel free to elaborate on any of your answers above.** (open-ended question)

***Talk-back session (Virtual Discussion)***

**29. Did you attend the talk-back session following the performance?**

- Yes
- No
- Partly
- No, but I would have liked to.

**30. If so, was it helpful to put the performance into context?**

- Yes
- No
- Somewhat
- Other : ______

**31. Please feel share any thoughts or comments resulting from the talk-back session.**

**32. We may hold future live or virtual events around this performance in the future. Please feel free to share what discussion topics or aspects of the performance you would like us to focus on for future events.**

***Show Notes and Supplementary Material***

**33. Do you plan to consult the notes and supplementary material?**

- Yes
- No
- Maybe
- I already have

**34. Please feel free to share any thoughts or comments regarding the supplementary material. We are still adding content, and would love to know what additional information you would find helpful.**

**3. *Parkinson’s* and *Dementia* pre-performance questionnaire (French)**

**1. Comment décririez-vous votre lien avec [la maladie de Parkinson / démence] ? Veuillez cocher toutes les cases qui s'appliquent.**

- - Je suis atteint(e) de la maladie de Parkinson / une démence.
  - Je suis un membre de la famille d'une personne atteinte de la maladie de Parkinson / d'une démence.
  - Je suis l'ami(e) d'une personne atteinte de la maladie de Parkinson / d'une démence.
  - Je suis un(e) étudiant(e) qui effectue des recherches sur la maladie de Parkinson / la démence.
  - Je suis professeur(e) qui effectue des recherches sur la maladie de Parkinson ou la démence.
  - Je suis un(e) professionnel(le) de la santé qui s'occupe de personnes atteintes de la maladie de Parkinson / d'une démence.
  - Je suis un(e) thérapeute complémentaire s'occupant de personnes atteintes de la maladie de Parkinson ou de démence.
  - Je n'ai aucun lien avec la maladie de Parkinson ou la démence.
  - Autre : ______

**2. Lequel décrit le mieux votre genre?**

- Homme
- Femme
- Non-binaire
- Autre : _______
- Préfère ne pas répondre

**3. Quel est votre âge?**

- < 20
- 20 – 35
- 35 - 50
- 50 – 65
- 65 – 80
- > 80
- Préfère ne pas répondre

**4. Est-ce que vous comprenez le français et l'anglais ?**

- - Français seulement
  - Oui, mais je préfère le français
  - Oui, mais je préfère l’anglais
  - Les deux langues, également

**5. Sur une échelle de 1 à 7, à quel point pensez-vous comprendre l'expérience vécue de la maladie de Parkinson / démence ?**

1 = Pas du tout
2 = Mal
3 = Assez mal
4 = Moyen
5 = Assez bien
6 = Très bien
7 = Complètement

**6. Sur une échelle de 1 à 7, à quel point pensez-vous comprendre la recherche scientifique sur la maladie de Parkinson / démence ?**

1 = Pas du tout
2 = Mal
3 = Assez mal
4 = Moyen
5 = Assez bien
6 = Très bien
7 = Complètement

**7. Veuillez nous partager en quelques phrases pourquoi vous êtes intéressé.e par ce spectacle et ce que vous espérez en retirer.**

**4. *Parkinson’s* and *Dementia* post-performance questionnaire (French)**

***Questions ouvertes***

**1. Quels sont les messages clés que vous avez retenus du spectacle ?**

**2. Quels sentiments le spectacle a-t-il suscité en vous ? Comment l'avez-vous vécu ?**

**3. Quels étaient les points forts du spectacle ?**

**4. Quelles étaient les faiblesses du spectacle ?**

**5. Ce spectacle changera-t-il votre façon de penser ou d'agir à l'égard de la maladie de Parkinson / démence ? Comment ?**

**6. Pensez-vous qu'une performance multimédia est un format approprié pour s'informer à la fois sur l'expérience vécue et la recherche scientifique sur la maladie de Parkinson / démence ? Veuillez expliquer.**

***Appréciation du spectacle***

Veuillez utiliser l'échelle suivante pour répondre aux questions ci-dessous.
1 = Fortement en désaccord ; 2 = En désaccord ; 3 = Plutôt en désaccord ; 4 = Ni d'accord ni en désaccord ; 5 = Plutôt d'accord ; 6 = D'accord ; 7 = Fortement d'accord

**7. J'ai apprécié le spectacle.**

1 2 3 4 5 6 7

**8. J'ai trouvé que ce spectacle était une bonne utilisation de mon temps.**

1 2 3 4 5 6 7

**9. J’ai apprécié la qualité artistique de ce spectacle.**

1 2 3 4 5 6 7

**10. Ce spectacle m'a interpellé sur le plan émotionnel.**

1 2 3 4 5 6 7

**11. Ce spectacle m'a interpellé sur le plan intellectuel.**

1 2 3 4 5 6 7

**12. Je recommanderais ce spectacle à d'autres personnes.**

1 2 3 4 5 6 7

**13. Je serais intéressé(e) à assister à un événement similaire à l'avenir.**

1 2 3 4 5 6 7

**14. J’ai pu facilement suivre le contenu du spectacle malgré le format bilingue.**

1 2 3 4 5 6 7

**15. N'hésitez pas à développer l'une de vos réponses ci-dessus.** (question ouverte)

***L’expérience vécue***

Veuillez utiliser l'échelle suivante pour répondre aux questions ci-dessous.
1 = Fortement en désaccord ; 2 = En désaccord ; 3 = Plutôt en désaccord ; 4 = Ni d'accord ni en désaccord ; 5 = Plutôt d'accord ; 6 = D'accord ; 7 = Fortement d'accord

**16. Le spectacle a amélioré ma compréhension de l'expérience subjective de la maladie de Parkinson / démence.**

1 2 3 4 5 6 7

**17. Le spectacle m'a aidé à mieux comprendre les problèmes émotionnels et psychologiques associés à la maladie de Parkinson / démence.**

1 2 3 4 5 6 7

**18. Le spectacle m'a amené à réfléchir à des questions liées à la maladie de Parkinson ou à la démence d'une manière nouvelle.**

1 2 3 4 5 6 7

**19. Le spectacle a augmenté mon empathie envers les personnes atteintes de la maladie de Parkinson / de démence.**

1 2 3 4 5 6 7

**20. Je pense que mon comportement envers les personnes atteintes de démence / maladie de Parkinson changera à la suite de ce spectacle.**

1 2 3 4 5 6 7

**21. Le spectacle a été un moyen utile de mieux connaître l'expérience vécue de la maladie de Parkinson / démence.**

1 2 3 4 5 6 7

**22. N'hésitez pas à développer l'une de vos réponses ci-dessus.** (question ouverte)

***Communication scientifique***

**23. J'ai appris quelque chose de nouveau sur la science concernant la maladie de Parkinson / démence.**

1 2 3 4 5 6 7

**24. Le spectacle a suscité mon intérêt pour la recherche scientifique sur la maladie de Parkinson / démence.**

1 2 3 4 5 6 7

**25. L'utilisation des arts du spectacle me fait mieux comprendre la pertinence de la recherche (ex. diagnostiques, causes neurologiques, etc.)**

1 2 3 4 5 6 7

**26. Le spectacle a été un moyen utile de découvrir la recherche sur la maladie de Parkinson / démence.**

1 2 3 4 5 6 7

**27. Je préfère apprendre les résultats de la recherche par le biais d'une performance que par une diffusion plus conventionnelle des connaissances académiques (ex. conférence, article scientifique).**

1 2 3 4 5 6 7

**28. N'hésitez pas à développer l'une de vos réponses ci-dessus.** (question ouverte)

***Discussion virtuelle avec le public***

**29. Avez-vous assisté à la séance de discussion qui a suivi le spectacle ?**

- Oui
- Non
- En partie
- Non, mais j’aurais aimé

**30. Si oui, cela vous a-t-il aidé à mettre le spectacle dans son contexte ?**

- Oui
- Non
- Un peu
- Autre : __________

**31.** **Il se peut que nous organisions à l'avenir des événements en direct ou virtuels autour de ce spectacle. N'hésitez pas à nous faire part des sujets de discussion ou des aspects du spectacle sur lesquels vous souhaiteriez que nous nous concentrions lors de futurs événements.**

**32. Veuillez nous faire part de toute réflexion ou de tout commentaire résultant de la séance de discussion*.***

***Notes du spectacle et matériel supplémentaire***

**33. Avez-vous l'intention de consulter le matériel supplémentaire?**

- Oui
- Non
- Peut-être
- Je l'ai déjà fait

**34. Si vous avez déjà visité la galerie, cela vous a-t-il aidé à mettre la performance dans son contexte ?**

**35. Veuillez nous faire part de toute réflexion ou commentaire résultant de la visite de la galerie.** **Nous continuons à ajouter du contenu, et nous aimerions savoir quelles informations supplémentaires vous trouveriez utiles.**
